# Changes in the Type of Sports Activity Due to COVID-19: Hypochondriasis and the Intention of Continuous Participation in Sports

**DOI:** 10.3390/ijerph17134871

**Published:** 2020-07-06

**Authors:** Chulhwan Choi, Chul-Ho Bum

**Affiliations:** 1 Department of Golf Industry, College of Physical Education, Kyung Hee University, Seocheon-dong 1, Giheung-gu, Yongin-si, Gyeonggi-do 17104, Korea; chulhwan.choi@khu.ac.kr; 2 Department of Sports Bigtainment, Graduate School of Physical Education, Kyung Hee University, Seocheon-dong 1, Giheung-gu, Yongin-si, Gyeonggi-do 17104, Korea

**Keywords:** type of sports activity, COVID-19, hypochondriasis, intention of continuous participation, social distancing, quarantine

## Abstract

This study focused on empirically analyzing sport activity participants’ perceptions of hypochondriasis caused by fear of infection and changes in continuous participatory behavior patterns. To this end, a comparative analysis was conducted with a focus on the forms of participation and age of sport activity participants. For the final comparative analysis, a 2 × 3 factorial multivariate analysis of variance was conducted after confirming the validity and reliability of data based on 229 questionnaires collected from healthy respondents who have never been infected with COVID-19. The results showed statistically significant differences between worry about illness, which is a subscale of hypochondriasis, disease phobia, thanatophobia, and intent to continue according to age. Statistically significant differences were also found when considering the type of sport for worry about illness, which is a subscale of hypochondriasis, disease phobia, symptom preoccupation, and intent to continue. Furthermore, interaction effects between the two independent variables, i.e., age and type of sport, were found for disease phobia, a subscale of hypochondriasis, and thanatophobia. In summary, age and type of sport, which are important factors for the COVID-19 infection and symptoms, were found to affect the sport activity patterns. These results proved that COVID-19 may have caused the participants to have different perceptions depending on their characteristics and change their form of continuous participation. These findings will provide useful data for predicting the perception and behavioral patterns of sports participants if diseases like COVID-19 occur in the future. They also show how to live a healthy life through exercise.

## 1. Introduction

### 1.1. Background

According to Sato, Jordan, and Funk [1], there is an increased interest in healthy living in contemporary society, as well as an effort to attain it. In particular, the work–life balance, which has taken center stage recently, implies that people can control and adjust their lives by themselves and that they can reach a state of satisfaction with their lives by properly distributing psychological and physical energy between their work and leisure [2]. Such a work–life balance is a universal concept necessary to attain an enjoyable life for all classes of people (i.e., men, women, married, unmarried, the youth, and the elderly), and it occurs in various dimensions (e.g., commitment to and reward of work, as well as health and safety, human relations, and stress management) [3]. In this social climate, leisure activities or outdoor physical activities are considered as very important activities that can lead to one’s physical health, as well as psychological stability [4,5]. Recently, however, external or environmental threats not encountered before have been increasing in number and frequency.

Recently, respiratory viruses have started to pose a mortal threat to the health of people around the globe, and it is highly likely that they will continue to pose a threat to humanity. Specifically, respiratory syndromes such as severe acute respiratory syndrome (SARS) or Middle East respiratory syndrome (MERS) have been encountered in the past, and in 2019, a different type of respiratory syndrome called coronavirus disease of 2019 (COVID-19) is paralyzing the world [6]. So far, because it is known that COVID-19 is transmitted through droplets or personal contact, prevention guidelines for sneezing and touching contaminated objects should be strictly observed [7]. Generally, COVID-19 has an incubation period of 1–14 days, and it shows various respiratory symptoms such as fever, cough, dyspnea, and pneumonia, which can range from mild to severe in severity [8]. Vaccines are currently being developed, and the fatality rate is commonly known to be between 1% and 2%. However, death is more frequent in the case of patients with underlying diseases, compromised immunity, or advanced age [9].

Ultimately, the World Health Organization (WHO) declared the COVID-19 pandemic in March 2020 [10]. That is, the COVID-19 pandemic is judged globally to be in the highest risk phase among pandemic alert phases. The state of emergency was declared in almost all regions, including Asia, North America, and Europe, and due to a sharp increase in the number of confirmed cases of infection and death, administrative orders were issued to temporarily shut down all facilities except the essential ones, such as hospitals and grocery stores, and national infrastructure that supplies electricity or water [11]. People have been advised to refrain from outside activities and isolate themselves at home, and practice social distancing that minimizes contact with others [12].

People are anxious about the possibility of infection by the highly contagious virus despite various social codes of conduct set to prevent its spread. The psychological anxiety of becoming infected by someone in situations where total self-isolation is impossible due to the reality also affects the lives of most healthy people [13]. Researchers have considered hypochondriasis as a type of physical disorder, but Rachman [14] has recently contended that it is desirable to understand it as excessive anxiety related to disease. That is, hypochondriasis is conceptualized as a type of psychological anxiety disorders. The reason is that the common psychological changes in hypochondriasis, such as severe anxiety and over-interpretation of minor physical symptoms, are also frequently observed in typical anxiety disorders [15]. Summarizing previous studies, it appears to be appropriate to consider hypochondriasis as a syndrome due to interactions among complex symptoms rather than one symptom [16]. It may be valid to consider the conceptualizations of hypochondriasis as complementary explanations of the various aspects of hypochondriasis that are difficult to define by one word rather than as being exclusive to each other [17]. The reason for selecting hypochondriasis as an important factor in the present study was that the epidemic of infectious viruses such as COVID-19 after SARS and MERS will continue to occur in the future, and research on the majority of the citizens who are currently healthy but experience anxiety about infection is required as much as the research on core subjects, such as the effective policies of government agencies that are responsible for developing therapeutic agents and controlling diseases.

The variable used as a key factor in the present study was the intention of continuous participation. This was done due to the fact that most people continue their everyday lives because the government did not enforce measures such as mandatory travel restrictions despite the widespread fear or anxiety caused by COVID-19. In particular, daily physical or leisure activities not only minimize psychological damages such as hypochondriasis as pointed out above but also improve the immune system that fights against viruses [18]. There are people who enjoy personal exercise that does not require contact with other people and who practice social distancing, while there are others who continue to participate in group exercises like before, and it was deemed meaningful to analyze whether there were changes in the life patterns in a situation where COVID-19 continues to spread. It is known that the intention of continuous participation arises from positive psychological outcomes such as pleasure, joy, and satisfaction due to participation [19]. There are previous studies [18,20] reporting that physical activities help maintain positive psychological state and reduce stress or anxiety, but group activities in which contact with others occurs are extremely restricted in special situations where COVID-19 is prevalent. Consequently, analyzing changes that occur in the behavior of people who currently participate in various types of sports activities may contribute to foreseeing and preparing for possible future epidemics.

### 1.2. Objectives

In the case of South Korea where the present study was conducted, the number of confirmed cases sharply increased after the first confirmed case on 20 January 2020, but currently, there is a decrease recorded [21]. However, psychological anxiety and concerns are increasing not only due to limitations in outdoor activities, but also physical and leisure activities by voluntary social distancing and compliance with self-isolation rules for an extended period. In addition, because COVID-19 is transmitted through droplets or personal contact, physical activities are limited, which resulted in changes in the leisure patterns of people. Accordingly, the present study analyzes the health concerns caused by COVID-19 and the intention of people to continue to participate in sports. Specifically, age (i.e., young, middle-aged, older adults) and type of sport (individual and group sport activities), which may act as the most important variables for the COVID-19 infection, were set as independent variables, and hypochondriasis and the intention of continuous participation were set as dependent variables. The present study is considered to be an important research attempt that analyzes and predicts the behavior of people not only in COVID-19 but also future pandemics because of the continuous outbreaks of respiratory syndromes.

## 2. Materials and Methods

### 2.1. Study Design

Data were collected from 10 March to 6 April 2020 in the Han River National Park and from three large sports centers located in Seoul. The survey was conducted when the whole nation became aware of the seriousness of COVID-19 in that the number of confirmed cases sharply increased after the first confirmed case on 20 January 2020, and on April 6 when the data collection was terminated, there were 10,284 accumulated confirmed cases: 6598 full recoveries, 3500 confirmed cases receiving medical care, and 186 deaths. Before participating in the survey, participants were asked if they were currently diagnosed with COVID-19 or had been previously diagnosed but completely cured, and those participants who had been infected were excluded. Subjects were healthy individuals (aged > 20 years) who had been participating in physical activities continuously, and the questionnaire was completed on a voluntary basis. In this study, physical activity has been defined as any bodily movement resulting in energy expenditure [22]. Furthermore, subjects were informed before their participation that there was no advantage or disadvantage in the survey participation and that they could stop participating any time. It took about 10 min to complete the questionnaire, and no sensitive personal information was collected.

### 2.2. Participants

A total of 300 questionnaires were distributed, and 231 were collected (~77% response rate), of which 229 responses were used in the statistical analysis after excluding incomplete or insincere responses. The participants of the 229 collected responses were grouped according to the two independent variables of age and type of sport used in the present study. First, subjects were grouped into young, middle-aged, and older adults by their age depending on guidelines [23] for the classification of age group cutoffs. At the same time, they were divided into two groups of individual and group sports, depending on the type of sport they participated in. In addition, the participants answered basic questions such on their gender and the frequency of recent sport participation. The results of descriptive statistical analysis of the participants in each group are presented in Table 1.

### 2.3. Instruments

The scale of hypochondriasis, which was used as the dependent variable in the present study, was based on the Illness Attitude Scale (IAS) developed by Kellner [24]. The factor structure of the scale was confirmed by Yi [25], based on the illness attitude of college students. The scale was revised and supplemented in the study by Moon [17], who investigated hypochondriasis from a psychological perspective and revised and supplemented the material again for the present study. Specifically, previous studies used nine subscales, while the present study uses four subscales of (a) thanatophobia, (b) worry about illness, (c) disease phobia, and (d) symptom preoccupation, which are appropriate to analyze hypochondriasis in COVID-19.

For the other dependent variable in the present study, intention of continuous participation, the measuring methods of Courneya and McAuley [26], Poff [27], and Jeon, Lee, and Kim [28] were consulted. Their methods were revised and supplemented for the purpose of the present study and reconstructed with four items for a single factor. All scales discussed above were on a 5-point Likert scale, ranging from 1 point for “strongly disagree” to 5 points for “strongly agree.”

## 3. Results

### 3.1. Scale Validity and Reliability

The scales used in the present study have already been tested in previous studies, and their validity and reliability were satisfactory. Exploratory factor analysis was performed before the full-scale analysis because the scales revised and supplemented for the present study were different, in addition to the research topic, purpose, and target population. Specifically, excluding intention of continuous participation, which was used as a single-factor dependent variable, factors with eigenvalue 1.00 or greater were selected using a Varimax rotation to determine the factor structure of hypochondriasis of people in a situation where COVID-19 is widespread. A total of four factors were selected, and details are presented in Table 2.

Additionally, reliability between the items was tested based on Cronbach’s Alpha coefficients, and a Cronbach’s alpha > 0.7 was considered satisfactory [29]: (a) worry about illness, α = 0.813; (b) disease phobia, α = 0.784; (c) symptom preoccupation, α = 0.787; (d) thanatophobia, α = 0.817; and (e) intention of continuous participation, α = 0.857. The results showed that all scales used in the present study had satisfactory statistical reliability.

### 3.2. Factorial Multivariate Analysis of Variance: Differences of Age and Type of Sport on Hypochondriasis and Intention of Continuous Participation

A 2 × 3 factorial multivariate analysis of variance (MANOVA) was performed to investigate the main and interaction effects of the two independent variables, age (i.e., young, middle-aged, and older adults) and type of sport (i.e., individual and group activities) on five dependent variables (i.e., four factors of hypochondriasis and a factor of intention of continuous participation).

The Wilks’ Λ multivariate F statistic found the statistically significant main effect of age on the dependent variables [Wilks’ Λ = 0.496, F(10, 438) = 18.386, *p* < 0.05]. Specifically, the univariate tests for worry about illness, F(2, 223) = 25.522, *p* < 0.05, disease phobia, F(2, 223) = 12.868, *p* < 0.05, thanatophobia, F(2, 223) = 8.016, *p* < 0.05, and intention of continuous participation, F(2, 223) = 54.736, and *p* < 0.05 based on age (i.e., young, middle-aged, and older adults) were found to be statistically significant. Detailed statistic results of the main effect and mean scores of each factor by groups have been described in Table 3.

As discussed above, statistically significant differences were found among the three age groups. To determine which groups were significantly different, a post hoc analysis was performed. First, among the four variables that showed statistically significant differences, which were (a) worry about illness, (b) disease phobia, (c) thanatophobia, and (d) intention of continuous participation, higher scores were obtained in relatively higher age groups of middle-aged (G2) and older adults (G3) than young adult group for worry about illness and disease phobia variables. In the case of thanatophobia, higher average values were obtained for the older adult group (G3) than the other two groups. Lastly, the intention of continuous participation variable showed that the younger the age group, the higher the intent of continuous participation. The statistical results of post-hoc analyses are presented in Table 4, and mean values between groups of the dependent variables are shown in Table 3.

Yet another main effect of the type of sport on five dependent variables was also statistically significant [Wilks’ Λ = 0.494, F(5, 219) = 44.924, *p* < 0.05]. Additionally, the univariate tests found there were statistically significant differences between Worry about Illness, F(1, 223) = 183.419, *p* < 0.05, disease phobia, F(1, 223) = 8.399, *p* < 0.05, symptom preoccupation, F(1, 223) = 12.680, *p* < 0.05, and intention of continuous participation, F(1, 223) = 26.822, *p* < 0.05, depending on the type of sport (i.e., individual and group activities). Detailed statistic results of the main effect and mean scores of each factor by groups have been described in Table 5.

Lastly, the results showed statistically significant two-way interaction effect of age and type of sport on five dependent variables [Wilks’ Λ = 0.832, F(10, 438) = 4.210, *p* < 0.05]. Specifically, the test indicated that the interaction effects of age and the type of sport were statistically significant for disease phobia, F(2, 223) = 5.314, *p* < 0.05, and thanatophobia, F(2, 223) = 13.200, *p* < 0.05. Detailed statistic results of interaction effects have been described in Table 6, and the mean scores of each variable by groups have been reported in Table 7.

**Figure 1:**
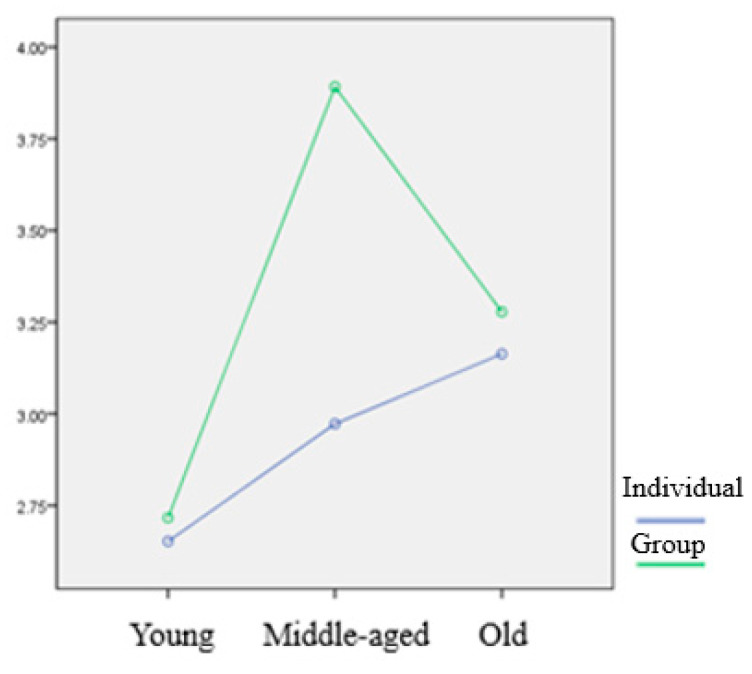
Interaction effects of Disease Phobia.

**Figure 2:**
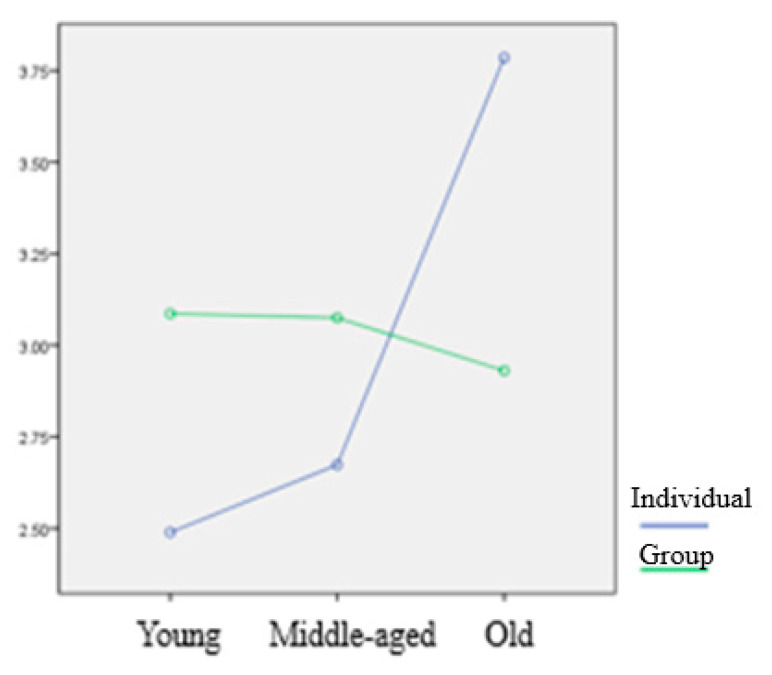
Interaction effects of Thanatophobia.

As discussed above, a statistically significant two-way interaction effect of age and type of sport was found for the factors of disease phobia and thanatophobia, and detailed differences between the variables are presented in Figure 1 and Figure 2. Similar interaction effects were found for both factors, and the results in the present study showed that the higher the age, the higher the scores of disease phobia and thanatophobia, while the respondents in the older adult group who participated in group sport activities showed low scores of disease phobia and thanatophobia.

## 4. Discussion

Currently, COVID-19, which is known to transmit through the respiratory system, not only threatens human lives, but also puts national systems around the world at risk due to its rapid spread through personal contact. Although social distancing is encouraged, long-term self-isolation has been difficult in some cases [30]. People are gradually attempting outside activities for minimum daily living, especially outdoor sport activities by which people can strengthen the immune system and obtain psychological stability. People who are infected with the virus are treated in isolation, but most healthy citizens experience higher levels of anxiety of being infected along with the interruption of daily life [31]. Therefore, the present study attempts to analyze the psychological state and changes in the pattern of sports participation in the coronavirus pandemic by analyzing the COVID-19 health concerns of citizens who continue their everyday leisure activities and their intent to continue to participate in leisure activities. In particular, considering the experience from the outbreak of MERS and SARS in the past and the current COVID-19, the findings of the present study are judged to be useful as a preparation for future uncertainties in a situation where the eradication of the virus is uncertain.

Based on the current released statistics, COVID-19 infection has shown higher fatality rates in the elderly. In fact, the mortality in the elderly is ≥30% according to the statistics of the Korea Centers for Disease Control and Prevention announced on April 24, which differs greatly than the death rate of about 1% in people < 60 years [9]. Thus, the present study analyzed the data using age as one of the important independent variables for classifying the respondents. The results of the present study showed that the higher the age, the higher the score for three of the four factors of hypochondriasis. Specifically, a higher level of worries and fear for health was found in the two older age groups (i.e., middle-aged and older age groups) compared to the young age group for the two factors of worry about illness and disease phobia. In addition, higher scores were found for the older age group, which is the oldest age group, for thanatophobia than the other two groups. This finding is consistent with the fatality rate announced by the Korea Centers for Disease Control and Prevention. Government’s real-time announcement of the infections and deaths by COVID-19 may cause fear of death in the elderly.

This finding of the present study is consistent with the interpretation of hypochondriasis as excessive anxiety related to diseases and conceptualization as a type of psychological anxiety disorders in recent studies [14,15,16]. In particular, in the case of highly contagious COVID-19, psychological anxiety may be higher because infected persons cannot be identified by the naked eye. The lower scores of the young age group for all factors statistically support the explanation that age is statistically a very important factor for the hypochondriasis in COVID-19. Furthermore, lower age groups answered that they will continue their current leisure activities even for the question of continued participation in leisure activities. The will itself to participate in leisure activities is absolutely the matter of personal choice since leisure activities following the infection rules are not regulated. In addition, no significant statistical difference was found in symptom preoccupation, which is the last factor of hypochondriasis, by age, and it may be attributable to the role of safe and rapid diagnostic tests performed by the South Korean government. Since rapid diagnostic tests are available for those experiencing mild symptoms at community health centers nearby, citizens’ vague concerns about their symptoms appear to be reduced regardless of their age.

Next, the present study compared and analyzed hypochondriasis and intent to continue according to the type of sports activity continued by the questionnaire respondents. As discussed above, COVID-19 is known to spread through contact between people [9]. Accordingly, the type of sport is important when desiring to participate in a sports activity. Basically, keeping a 1–2-m distance from others is recommended [7], but such social distancing is practically impossible in the case of group sports. Of course, gathering of a large number of spectators such is the case when watching professional sports is discouraged, but the participation itself is possible because its restriction has not been enforced for specific sports. Accordingly, Type of Sports Activity was used as an important independent variable that classifies questionnaire respondents.

Consequently, respondents who answered that they participated in group sports showed relatively higher scores than respondents who participated in individual sports considering worry about illness, disease phobia, and symptom preoccupation among the four factors of hypochondriasis. This appears to indicate that the psychological anxiety of people who participate in group sports is voluntary. The score of group sport participants in intent to continue was on the contrary low considering the results of hypochondriasis. These responses indicate that the individuals currently participate in group sports but are anxious about their health and that they intend to discontinue group sports activities in the future. It is understood that these individuals are slightly anxious when it comes to the possibility of infection due to the rapid spread of COVID-19, and their will not to participate in group sports anymore is shown. Participating in social movements is considered to be a positive response to overcoming the virus.

Lastly, a meaningful phenomenon was found in the results of the interaction effect of factorial MANOVA performed in the present study. We analyzed the influence of the interaction between the two independent variables (i.e., age and type of sport) on the five dependent variables (four hypochondriasis subscales and one intent to continue). The results showed that there were interaction effects between age and type of sport on two dependent variables (i.e., disease phobia and thanatophobia). The interaction showed that the higher the age, the score of disease phobia and thanatophobia increased, but in the case of respondents who were in the highest age group and participated in group sports, it showed relatively low scores. It means that the high fatality rate in the elderly generally tended to increase the anxiety of the older age group, but the tendency was not found only in the elderly who participated in group sports. In the case of golf, which was one of the group sports examined in the present study, psychological awareness appeared to be lowered because a contact was made with a limited number of people, and it is played outdoors. It appears to be, however, far from the social movements that basically minimize contact with others and violate the rules. Therefore, it should be kept in mind that the awareness must be maintained until the end of pandemic.

## 5. Conclusions and Limitations

There is now increased public knowledge that sports participation entails not only physical benefits but also mental and social benefits, people’s interest in health has steadily increased. External factors that limit people’s participation in sports or change their participation patterns, however, have always existed. Among them, environmental factors have recently caused large changes in people’s participation in sports. COVID-19, which was rampant at the end of 2019 and rapidly spread through contacts with people and via the respiratory system, changed not only people’s daily lives but also sports participation patterns. The present study analyzed the hypochondriasis and sports participation types of people due to COVID-19 pandemic. Statistically significant results were derived by applying age and interpersonal contacts, which are important risk factors for COVID-19 infection, shown by medical statistics indicators. Changes in daily life by these viruses are expected to continue in the future as well, and it is hoped that the findings of the present study will be used as important reference data.

However, this study used convenience sampling to draw the samples, which can lead to certain restrictions in obtaining representativeness in research. Accordingly, it is necessary to take account of various sampling methods that use probabilistic sampling in further research. Moreover, with sport participants rapidly decreasing in number due to the spread of COVID-19, there were many difficulties in securing research participants for this study. Follow-up research must remedy these shortcomings. Finally, as this was not a clinical study but a social science study analyzing the psychological characteristics of people, we investigated whether the participants had “ever been infected with COVID-19” or “had suspicious symptoms” using a survey. Further research must produce more scientifically sound results through the convergence with clinical science.

## Figures and Tables

**Table 1:** Descriptive statistics by age and type of sport.

	Young		Middle-Aged		Older	
	Individual	Group	Individual	Group	Individual	Group
Gender						
Male	30 (66.7%)	11 (40.7%)	27 (55.1%)	25 (62.5%)	26 (59.1%)	12 (50.0%)
Female	15 (33.3%)	16 (59.3%)	22 (44.9%)	15 (37.5%)	18 (40.9%)	12 (50.0%)
Type of Sport						
Home training	13 (28.9%)	-	9 (18.4%)	-	8 (18.2%)	-
Walking	8 (17.8%)	-	10 (20.4%)	-	9 (20.5%)	-
Running	9 (20.0%)	-	8 (16.3%)	-	8 (18.2%)	-
Tracking	13 (28.9%)	-	13 (26.5%)	-	8 (18.2%)	-
Cycling	2 (4.4%)	-	9 (18.4%)	-	11 (25.0%)	-
Golf	-	5 (18.5%)	-	10 (25.0%)	-	6 (25.0%)
Gym	-	5 (18.5%)	-	10 (25.0%)	-	6 (25.0%)
Basketball	-	5 (18.5%)	-	8 (20.0%)	-	4 (16.7%)
Baseball	-	9 (33.3%)	-	10 (25.0%)-		4 (16.7%)
Swimming	-	1 (3.7%)	-	4 (10.0%)	-	5 (20.8%)
Frequency of playing						
Rarely	10 (22.2%)	3 (11.1%)	9 (18.4%)	6 (15.0%)	12 (27.3%)	2 (8.3%)
Sometimes	8 (17.8%)	8 (29.6%)	9 (18.4%)	15 (37.5%)	9 (20.5%)	6 (25.0%)
Often	10 (22.2%)	6(22.2%)	13 (26.5%)	5 (12.5%)	8 (18.2%)	4 (16.7%)
Very Often	13 (28.9%)	7 (25.9%)	14 (28.6%)	10 (25.0%)	9 (20.5%)	6 (25.0%)
Always	4 (8.9%)	3 (11.1%)	4 (8.2%)	4 (10.0%)	6 (13.6%)	6 (25.0%)
Total	45 (100%)	27 (100%)	49 (100%)	40 (100%)	44 (100%)	24 (100%)

**Table 2:** Results of exploratory factor analysis and scale reliability.

Items	1	2	3	4
I fear when I hear news that reminds me of death.	**0.913**	0.126	0.045	0.034
Thinking about death makes me feel afraid.	**0.857**	0.019	0.013	−0.004
I fear that I might die.	**0.788**	−0.067	0.066	−0.153
I worry about health.	0.084	**0.882**	0.202	0.060
I worry about becoming seriously ill in the future.	−0.003	**0.837**	−0.016	0.003
I am scared when I think about serious diseases.	−0.004	**0.808**	0.215	0.101
I fear that I may contract COVID-19.	0.073	0.123	**0.892**	0.038
I fear that I may become seriously ill.	0.156	0.077	**0.838**	0.011
When I read an article about virus, I feel similar symptoms.	−0.088	0.152	**0.737**	0.033
When a symptom persists, I believe I have a serious illness.	−0.123	0.023	0.004	**0.895**
If I detect a symptom, I can’t think anything else because of it.	0.030	0.079	−0.096	**0.836**
If I feel unusual symptoms in my body, I worry about them.	−0.035	0.046	0.180	**0.770**
Eigenvalues	2.992	2.411	1.816	1.548
Variance (%)	24.935	20.095	15.136	12.899

Note. 1 = Thanatophobia, 2 = Worry about Illness, 3 = Disease Phobia, 4 = Symptom Preoccupation.

**Table 3:** Results of main effect (age) on dependent variables.

Source	Dependent Variables	*df*	*F*	*p*		*Mean*	
Age	Hypochondriasis				G1	G2	G3
	Worry about Illness	2	25.522	0.000 *	2.491	**3.254**	**3.126**
	Disease Phobia	2	12.868	0.000 *	2.676	**3.390**	**3.202**
	Thanatophobia	2	8.016	0.000 *	2.713	2.856	**3.488**
	Symptom Preoccupation	2	0.490	0.613	3.153	3.167	3.005
	Intention of Continuous Participation	2	54.736	0.000 *	**3.656**	**2.901**	2.116

Note. * *p* < 0.05; G1 = young adult group, G2 = middle-aged adult group, G3 = older adult group; statistically significant higher mean scores among groups are in bold.

**Table 4:** Results of post hoc analyses: Differences on dependent variables by age groups.

		Worry about Illness	Disease Phobia	Thanatophobia	Symptom Preoccupation	Intention of Continuous Participation
G1	G2	0.000 *	0.000 *	0.563	0.995	0.000 *
	G3	0.000 *	0.002 *	0.000 *	0.606	0.000 *
G2	G1	0.000 *	0.000 *	0.563	0.995	0.000 *
	G3	0.454	0.418	0.000 *	0.518	0.000 *
G3	G1	0.000 *	0.002 *	0.000 *	0.606	0.000 *
	G2	0.000 *	0.418	0.000 *	0.518	0.000 *

Note. * *p* < 0.05; G1 = young adult group, G2 = middle-aged adult group, G3 = older adult group.

**Table 5:** Results of main effect (type of sport) on dependent variables.

Source	Dependent Variables	*df*	*F*	*p*	*Mean*	
Type of Sport	Hypochondriasis				G1	G2
	Worry about Illness	1	183.419	0.000 *	2.473	**3.736**
	Disease Phobia	1	8.399	0.004 *	2.930	**3.381**
	Thanatophobia	1	0.159	0.690	2.976	3.040
	Symptom Preoccupation	1	12.680	1.000 *	2.944	**3.370**
	Intention of Continuous Participation	1	26.822	0.000 *	**3.127**	2.560

Note. * *p* < 0.05; G1 = individual sport activity participation group, G2 = group sport activity participation group, G3 = older adult group; statistically significant higher mean scores among groups are in bold.

**Table 6:** Results of interaction effects of independent variables on dependent variables.

Source	Dependent Variables	*df*	*F*	*p*
Age * Type of Sport	Hypochondriasis			
	Worry about Illness	2	1.106	0.333
	Disease Phobia	2	5.314	0.006 *
	Thanatophobia	2	13.200	0.000 *
	Symptom Preoccupation	2	1.901	0.152
	Intention of Continuous Participation	2	0.033	0.968

Note. * *p* < 0.05.

**Table 7:** Mean scores for interaction effects.

Age	Type of Sport	WI	DP	TH	SP	ICP
**Young**	**Individual**	2.096	2.652	2.489	2.889	3.889
	**Group**	3.148	2.716	3.086	3.593	3.269
**Middle-aged**	**Individual**	2.632	2.972	2.674	3.104	3.156
	**Group**	4.000	3.892	3.075	3.242	2.594
**Older**	**Individual**	2.682	3.163	3.785	2.830	2.333
	**Group**	3.959	3.278	2.931	3.333	1.708

Note. WI, Worry about Illness; DP, Disease Phobia; TH, Thanatophobia; SP, Symptom Preoccupation; ICP, Intention of Continuous Participation; statistically significant higher mean scores among groups are in bold.
